# Flexible learning in complex worlds

**DOI:** 10.1093/beheco/arad109

**Published:** 2023-12-29

**Authors:** Olof Leimar, Andrés E Quiñones, Redouan Bshary

**Affiliations:** Department of Zoology, Stockholm University, 106 91 Stockholm, Sweden and; Institute of Biology, University of Neuchâtel, Emile-Argand 11, 2000 Neuchâtel, Switzerland; Institute of Biology, University of Neuchâtel, Emile-Argand 11, 2000 Neuchâtel, Switzerland

**Keywords:** Autostep, learning set formation, meta learning, prediction error, Rescorla-Wagner learning, reversal learning, stochasticity, volatility

## Abstract

Cognitive flexibility can enhance the ability to adjust to changing environments. Here, we use learning simulations to investigate the possible advantages of flexible learning in volatile (changing) environments. We compare two established learning mechanisms, one with constant learning rates and one with rates that adjust to volatility. We study an ecologically relevant case of volatility, based on observations of developing cleaner fish *Labroides dimidiatus* that experience a transition from a simpler to a more complex foraging environment. There are other similar transitions in nature, such as migrating to a new and different habitat. We also examine two traditional approaches to volatile environments in experimental psychology and behavioral ecology: reversal learning, and learning set formation (consisting of a sequence of different discrimination tasks). These provide experimental measures of cognitive flexibility. Concerning transitions to a complex world, we show that both constant and flexible learning rates perform well, losing only a small proportion of available rewards in the period after a transition, but flexible rates perform better than constant rates. For reversal learning, flexible rates improve the performance with each successive reversal because of increasing learning rates, but this does not happen for constant rates. For learning set formation, we find no improvement in performance with successive shifts to new stimuli to discriminate for either flexible or constant learning rates. Flexible learning rates might thus explain increasing performance in reversal learning but not in learning set formation, and this can shed light on the nature of cognitive flexibility in a given system.

## INTRODUCTION

The ability of animals to adjust to new and complex environments through learning is an important aspect of adaptive behavioral flexibility (Fawcett et al. 2013). In animal psychology and behavioral ecology, different meanings have been given to the terms behavioral or cognitive flexibility (Audet and Lefebvre 2017; Lea et al. 2020; Uddin 2021). Here, we are concerned with the ability to adjust to environmental change using learning, for instance, learning to select suitable food items. The question we ask is how well different learning rules (Fawcett et al. 2013), in the sense of different mechanisms of reinforcement learning, with either flexible (variable) or constant learning rates, serve to adapt behavior in a volatile (changing) environment. Specifically, we investigate how big the advantage of having flexible learning rates might be in a volatile environment.

It is known from neuroscience studies that humans and other animals adjust learning rates to the volatility of rewards (Behrens et al. 2007; Diederen and Schultz 2015; Grossman et al. 2022). An experimental example of volatility in rewards is reversal learning, where an individual first learns to discriminate between a rewarded and a non-rewarded option, and then the rewards are reversed, sometimes with successive episodes of reversal. Performance in such reversal learning is one measure that has been used to describe behavioral flexibility (Deaner et al. 2006; Bond et al. 2007; Liu et al. 2016; Izquierdo et al. 2017; Buechel et al. 2018; Boussard et al. 2021; Triki et al. 2022; Vardi and Berger-Tal 2022), and this performance might be improved by flexible learning rates. The reversal in rewards is a particular form of volatility, but it is similar to forms of volatility that may occur in nature, such as when bumblebees need to learn to associate a different flower color to floral rewards (Raine and Chittka 2012), or when birds need to learn to forage in a different tree species with seasonal change (Cauchoix et al. 2017). The performance in other measures of behavioral flexibility, such as learning set formation (also referred to as set-shifting), where an individual encounters a sequence of novel discrimination tasks (Harlow 1949; Wilson et al. 1985; Bailey et al. 2007; Audet and Lefebvre 2017), could conceivably also be enhanced by flexible learning rates. An example of set-shifting would be to discriminate complex stimuli first based on color, then based on shape, then based on pattern, etc. In an experiment with rats digging for food in bowls (Birrell and Brown 2000), first, the odor and then the medium in the bowl indicated which bowl contained food (as an example of the treatments used), and learning about similar shifts in which aspect of a stimulus is most relevant for reward might well occur in the wild.

Sometimes, a distinction is made between volatile and stochastic environments (Nassar et al. 2010; Soltani and Izquierdo 2019; Piray and Daw 2021; Topel et al. 2023). In volatile environments, there are changes over time in the expected (mean) outcome of choices and actions, whereas the term stochastic environments has been used to describe random variability in outcomes, with no changes in mean outcomes over time. Here, we are mainly concerned with volatility, but we also study flexible and constant learning rates in stochastic environments.

To investigate the potential advantages of flexible learning rates, we use a classical learning mechanism with constant rates as a baseline. Rescorla and Wagner (1972) introduced a model for classical conditioning with complex, multi-dimensional stimuli. The Rescorla-Wagner model can be extended to operant conditioning and is among the most investigated approaches to learning. The model updates the estimates of the value of each component or dimension of a complex stimulus (e.g., color, shape, presence/absence of a feature). Learning rates can differ between dimensions, but for a given dimension the rate is constant over time. While being a strong candidate for adaptive (optimal) learning, it is known that Rescorla-Wagner learning is not optimal (i.e., maximizing rewards obtained) in volatile environments (Dayan et al. 2000; Trimmer et al. 2012). Several alternatives to Rescorla-Wagner learning have been proposed, typically involving flexible learning rates. The basic idea is that high learning rates should be advantageous in volatile environments, where there is a need to learn to respond to changes, whereas low learning rates might be advantageous in stochastic environments. In our comparisons here, we model flexible learning rates using a learning algorithm called Autostep (Mahmood et al. 2012) because of its robustness in adapting learning rates without the need for extensive tuning of parameters. It is a refinement of the so-called delta-bar-delta algorithm (Jacobs 1988; Sutton 1992a, 2022), and it falls into the category of meta-learning approaches (Sutton 2022).

In the following, we outline the learning models and simulations we use, and then present results from different situations where flexible learning might be advantageous, comparing flexible (Autostep) with constant (Rescorla-Wagner) learning rates. The first situation is inspired by observations on developing cleaner fish *Labroides dimidiatus*. These fish typically occupy small territories (called “cleaning stations”), where so-called “client” fish visit to have ectoparasites removed (Côté 2000). In addition to ectoparasites, cleaner fish also consume the protective mucus layer on the clients’ scales, which leads to conflicts (Grutter and Bshary 2003). In nature, there are hundreds of client species, and they differ in ways that affect their value as a food source to cleaners, including variation in client body size, ectoparasite load, mucus quality, maneuverability, and aggressiveness (Grutter 1995; Bshary and Grutter 2002; Roche et al. 2021). These differences, as well as variation in species densities, influence how cleaner fish behave toward the client species (Bshary 2001; Binning et al. 2017; Triki et al. 2019, 2020). Juvenile cleaners interact with rather few client species (Triki et al. 2019), for which generally a larger body size indicates that the client is a better food patch (Grutter 1995), and we make use of this in our modeling. As cleaners grow and become adults, client species composition diversifies (Triki et al. 2019). For adult cleaners, relying solely on client size would lead to sub-optimal foraging decisions. Inspired by observations (Wismer et al. 2019; Roche et al. 2021), we investigate how incorporating additional client characteristics like color, body shape, and behavior could improve cleaner fish foraging performance.

The cleaner fish system illustrates challenges encountered by many learning animals. Examples include migrants experiencing a shift to a new and different foraging environment (Bairlein and Simons 1995; Pierce and McWilliams 2005) and seasonal changes that expose a forager to new food types (Janmaat et al. 2016). We also give an illustration of the performance of flexible and constant learning rates when the degree of stochasticity of the environment is high. Although the impacts of volatility versus stochasticity on learning have not been emphasized in experimental psychology or behavioral ecology, this has been dealt with in neuroscience (Nassar et al. 2010; Piray and Daw 2021). The general conclusion is that stochasticity should favor lower learning rates, allowing a learner to average over more trials.

We regard changes between environments, resulting either from individuals changing their activities, moving to new places, or encountering seasonal changes, as the most important type of environmental volatility in nature. Experimentally, however, paradigms such as reversal learning and learning set formation dominate the study of behavioral flexibility. For this reason, we also compare the performance of flexible and constant learning rates for a case of reversal learning, extended over several reversals, and we make a similar comparison for a case of learning set formation. These analyses could help behavioral ecologists decide which type of experiments are of relevance to their study species, based on the learning challenges that are met in nature.

Finally, we survey and comment on different models of flexible learning, in relation to previous and current ideas in experimental psychology (Mackintosh 1975; Pearce and Hall 1980; Pearce and Mackintosh 2010; Holland and Schiffino 2016; Soltani and Izquierdo 2019). We also discuss our results in relation to existing ideas about the nature of flexible learning, making the point that relatively simple mechanisms of adjustment of learning rates could, wholly or partially, explain some of the observed phenomena of flexibility of learning. Such adjustments could represent specific adaptations to environmental volatility, or they could be consequences of broader cognitive adaptations, for instance relating to attention and memory.

## LEARNING MODELS AND APPROACHES

The kind of learning we study is where an individual learns the values of stimuli that can be distinguished by certain characteristics, which we refer to as features of compound (multi-dimensional) stimuli. The characteristics define different stimulus dimensions, which can be things like the color, texture, or shape of potential food items encountered by a forager. For the cleaner fish example, a compound stimulus would be a client fish. The individual cleaner fish learns an estimate of a value for each feature of compound stimuli and uses the sum of these values to estimate the value of the client fish. We refer to this as “feature learning.” There are alternative ways that individuals might estimate the values of compound stimuli, for instance to form an entirely separate estimate for each type of stimulus. The latter is sometimes called “object learning” (Farashahi et al. 2020).

Here, we focus on feature learning. The approach corresponds to long-standing ideas about classical conditioning in experimental psychology, when animals respond to the component stimuli that are present in a learning trial. The case most frequently studied is that of absence/presence features (0/1 stimulus components), where a feature has only two states, either being absent or present in the compound, and we make use of this in our learning simulations. There are, of course, other cases, for instance, quantitative stimulus dimensions, and we include overall stimulus size as one such dimension.

Perhaps the most influential formulation of these ideas is the learning mechanism proposed by Rescorla and Wagner (1972). In their approach, if $w_{m}$ is an individual’s current estimate of the value of a certain absence/presence feature from stimulus dimension m, and the feature is present in a learning trial, the individual updates its estimate to $w_{m}^{\prime }$, where


$$\begin{array}{l} \begin{array}{l} w_{m}^{\prime }=w_{m}+\alpha_{m}\left(R-Q\right).\; \end{array} \end{array}$$ (1)

Here, R is the reward perceived by the individual from interacting with the compound, and Q is the individual’s previous estimate of the value of the compound. The quantity


$$\begin{array}{l} \begin{array}{l} \delta =R-Q\; \end{array} \end{array}$$ (2)

is referred to as the prediction error, and is the difference between the reward R currently experienced by the individual and its prior estimate Q of the reward. The change in the estimated feature value $w_{m}$ in equation (1) is thus the learning rate $\alpha_{m}$ times the prediction error, and tends to move the estimate toward the true value. Learning rates could differ between stimulus dimensions and could also change over time. The main question we ask is how big the advantage of flexible learning rates might be.

If $x_{m}$ indicates the feature from stimulus dimension m, so that $x_{m}$ is a 0/1 variable, the estimate of the value of the compound is given by the sum of all feature values that are present:


$$\begin{array}{l} \begin{array}{l} Q=\underset{m=1}{\overset{M}{\sum }}\;x_{m}w_{m}.\; \end{array} \end{array}$$ (3)

This formula also applies to quantitative stimulus dimensions, for which $w_{m}$ is the estimated reward per unit of the dimension. For simplicity, we limit ourselves to additive reward structures, although there are other cases that occur in nature, such as when features interact in indicating the value. There is also random variation in rewards. For instance, for client fish visiting cleaners, there is work showing that the number of ectoparasites that cleaners can feed on, corresponding to the value as a food patch, is correlated with client size, but the correlations are not extremely high (Grutter 1994, 1995).

**Figure 1:**
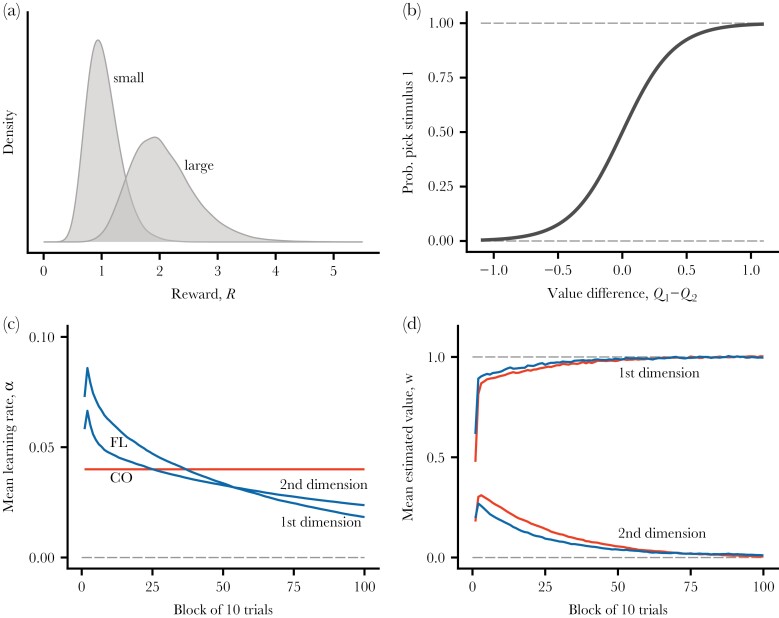
Overview of the first phase, where individuals learn to discriminate between two types of compound stimuli (“small” and “large” clients). (a) Distribution of rewards from the two types of compound stimuli. (b) The function from equation (4), giving the probability of choice from the difference in estimated values of the two compound stimuli present in a trial. (c) Average (over learning replicates) of flexible (FL) and constant (CO) learning rates for the two stimulus dimensions. (d) Average estimated values for flexible and constant learning rates for the two stimulus dimensions (first dimension has true value 1.0 and second has true value 0). There are 10 trials in a block and data are averages over 100 replicate learning simulations.

When an individual can choose between two compound stimuli with estimated values of $Q_{1}$ and $Q_{2}$, we assume that the individual chooses stimulus 1 with probability.


$$\begin{array}{l} \begin{array}{l} p_{1}=\frac{1}{1+exp\left(-\omega \left(Q_{1}-Q_{2}\right)\right)},\; \end{array} \end{array}$$ (4)

where ω is a parameter (we used ω=5. in our simulations; see the curve in Figure 1b below). This is referred to as a soft-max rule, going from estimated values to a choice, and it is commonly used in reinforcement learning models (Sutton and Barto 2018).

Rescorla and Wagner (1972) assumed that learning rates stay constant over time, but there are a number of suggestions for how they might vary. One idea is that an increase in prediction errors could indicate to an individual that it should change its learning rates. We will investigate how much better an individual with flexible learning rates is at selecting higher value compounds, compared to a Rescorla-Wagner learner.

The Autostep method (Mahmood et al. 2012) is a meta learning approach that adjusts learning rates based on the recent prediction-error history. An overall idea of such meta learning algorithms is to adjust learning rates in a way that minimizes prediction errors. Autostep is a further refinement of the incremental delta-bar-delta (IDBD) method (Sutton 1992a), making it more robust. The intuition behind IDBD is to increase a learning rate $\alpha_{m}$ if in recent trials the estimate $w_{m}$ has been increasing (decreasing) and the current prediction error indicates an additional increase (decrease) in $w_{m}$, corresponding to a positive correlation between the recent and current changes to $w_{m}$. Similarly, the learning rate is decreased for a negative correlation because this indicates that the current change in $w_{m}$ overshoots the true value. The algorithm also changes learning rates on the log-scale, which allows for a fairly large range of values for the rates. These properties of IDBD also hold for Autostep. More details on the learning algorithms we use appear in the supplements.

## LEARNING SIMULATIONS

As mentioned, the first learning environment we use in our simulations is inspired by the situation for developing cleaner fish, as they become adult and transition from a simpler to a more complex set of client fish species to choose between and clean. For the complex set, foraging efficiency can be increased by combining information about client size with information in other stimulus dimensions. Here, we describe simulations for such cases, where there is a transition from a simpler to a more complex learning environment.

### Stimulus dimensions and compound stimuli

In order to characterize many (up to 10) different compound stimuli (e.g., clients), there are 10 stimulus dimensions. The first four dimensions are as follows, together with their true values.

1. The first dimension, $x_{1}$, is quantitative (e.g., client size) and has a positive true value, $W_{1}=1.0$.
2. The second dimension, $x_{2}$, is 0/1, and has a zero true value, $W_{2}=0$, so it is an uninformative dimension (irrelevant for reward; a possible example is whether or not a client is colorful).
3. The third dimension, $x_{3}$, is 0/1 and has a positive true value, $W_{3}=1.0$, so it is an informative dimension (relevant for reward; a possible example is whether or not a client swims with pectoral fin movements, indicating a thicklip wrasse).
4. The fourth dimension, $x_{4}$, is 0/1 and has a negative true value, $W_{4}=-1.0$, so it is also a relevant dimension (a possible example is whether or not a client has a continuous second part of the dorsal fin, indicating a snapper, which has substances in the mucus making it less valuable).

An additional six 0/1 dimensions are described in Table 1 below. From combinations of the four first dimensions, we have four types of compound stimuli. These could correspond to four client species. Relevant criteria for variation in client value would be size, parasite load, mucus quality/quantity, and maneuverability (Roche et al. 2021). Of these, only size is directly visible to cleaners. Correlations between size and the other variables may exist but are weak enough that paying attention to features/dimensions other than size may help cleaners to improve their choices. We hence illustrate the scenario with four species/compound stimuli by considering size as a continuous variable, and colorfulness, swimming with pectoral fin, and a continuous second part of the dorsal fin as dichotomous (0/1).

**Table 1:** Characteristics of stimulus dimensions and compound stimuli used for the simulation of a change to a more complex world. There are 10 compound stimuli (CS1 to CS10) that can be distinguished using 10 stimulus dimensions. The first dimension represents size, with expected values small (1.0) and large (2.0), and the others are absence/presence (0/1) dimensions. The expected reward values per feature ($W_{m}$) are given in the second column and the features of the different compound stimuli are in the following columns.

Dim	Value	CS1	CS2	CS3	CS4	CS5	CS6	CS7	CS8	CS9	CS10
1	1.0	1.0	2.0	2.0	2.0	1.0	1.0	2.0	1.0	1.0	2.0
2	0.0	0	1	1	1	0	0	1	0	0	0
3	1.0	0	0	1	0	0	0	0	0	0	0
4	−1.0	0	0	0	1	0	0	0	0	0	0
5	2.0	0	0	0	0	1	0	0	0	0	0
6	1.0	0	0	0	0	0	1	0	0	0	0
7	−1.0	0	0	0	0	0	0	1	0	0	0
8	2.0	0	0	0	0	0	0	0	1	0	0
9	1.0	0	0	0	0	0	0	0	0	1	0
10	−1.0	0	0	0	0	0	0	0	0	0	1

1. The first type has small size, $x_{1}=exp\left(y_{small}+z_{x}\right)$, with $z_{x}$ normally distributed with mean zero and standard deviation $\sigma_{x}=0.25$, and $y_{small}$ so that ${\overset{↼}{\;x}}_{1}={\overset{↼}{x}\;}_{small}=1$ (which happens for $y_{small}=-\sigma_{x}^{2}/2$), and absence of features in the other dimensions. This could be a species of small clients, like less colorful damselfish.
2. The second type has large size, $x_{1}=exp\left(y_{large}+z_{x}\right)$, again with $z_{x}$ normally distributed with mean zero and standard deviation $\sigma_{x}$, and $y_{large}$ such that ${\overset{↼}{x}\;}_{1}={\overset{↼}{x}\;}_{large}=2$, and presence of a feature in the second dimension, and absence of features in the other dimensions. This could be a species of large clients that are characterized by a feature $x_{2}$ that is irrelevant for reward (size is sufficient to predict reward). An example could be a bream, for which being colorful contains no information about parasite load.
3. The third type of compound stimulus is the same as the second for the first two dimensions, but it has a feature present in the third dimension and no feature in the fourth. This is then a species of more valuable large clients. An example could be a thicklip wrasse, which is large, colorful and swims with pectoral fins. These fish are valuable food patches as they have particularly high parasite loads (Grutter 1995).
4. The fourth type of compound stimulus is the same as the second for the first two dimensions, and it has no feature in the third dimension but a feature present in the fourth. This is then a species of less valuable large clients. A snapper could be an example, being large, colorful, not swimming with pectoral fins, and having a continuous second part of the dorsal fin. These fish are less valuable as food patches as they have less preferred mucus (Grutter and Bshary 2004).

These compound stimuli, together with six additional compound stimuli, are described in Table 1. Note that we assume log-normal distributions for the first stimulus dimension and also for the stochasticity of rewards, in order to ensure that values are positive.

### Learning trials

We first consider two cases of sequences of learning trials. In both cases, there is an initial phase of *T* trials of learning (*T* = 1000) with only the first two compound stimuli in Table 1 (e.g., one species of small clients and one species of large clients). This is followed by a phase of an additional *T* trials of learning in a more complex world. In case 1, individuals learn to discriminate between the first four compound stimuli in Table 1 (which could be four client species). In case 2, the world is even more complex, such that individuals learn to discriminate all 10 compound stimuli in Table 1. In both cases, an individual can choose between two compound stimuli in each trial, and these are randomly drawn from all types that occur in that phase of learning of that case.

We also examine a case of reversal learning. In this simulation, there is first a phase of 100 trials where individuals can choose between a rewarded stimulus (*R* = 1), with a feature present in dimension 1, and a non-rewarded stimulus (*R* = 0), with no feature in dimension 1 but a feature present in dimension 2. In practice, the discrimination could be between blue and green stimuli. These 100 trials are enough for individuals to learn to prefer the rewarded stimulus. In the next 100 trials, the rewards are reversed. The entire procedure is then repeated for another 200 trials, that is, an additional two reversals.

Finally, we examine a case of learning set formation. In this simulation, the first 100 trials are as in the reversal learning case, but in subsequent intervals of 100 trials, entirely new pairs of rewarded and non-rewarded stimuli are used, with features in new stimulus dimensions. A total of four pairs are used, making up a total of 400 trials. As an example, the four pairs could be blue and green stimuli, followed by circular and square stimuli, followed by striped and plain stimuli, followed by horizontally and vertically oriented stimuli.

For the above cases, we present results based on replicate simulations of learning for 100 individuals. We assume that the reward from a compound stimulus has a log-normal distribution around the true expected value, with a standard deviation $\sigma_{R}$ on the log scale. For the transitions to a more complex world, we use $\sigma_{R}=0.10$ (in the supplements, we show results for higher stochasticity, $\sigma_{R}=0.50$; cases 3–6), and for reversal learning and learning set formation, we use $\sigma_{R}=0.02$.

As the starting value of learning rates, we use $\alpha_{m}=0.04$, which allows for learning of unit value differences over 50–100 trials. For the starting estimated values, we used $w_{m}=0$; this might hold for individuals without any previous experience of the stimulus dimension.

## RESULTS

### Transitions to a complex world

In the first phase of learning for cases 1 and 2, there are only two types of compound stimuli, which is illustrated in Figure 1. The variation in rewards, shown in Figure 1a, comes both from random variation in the first stimulus dimension (e.g., client size), and from random variation in rewards from a client with a given true expected reward. The sigmoid soft-max curve from equation (4) appears in panel b, and the learning rates $\alpha_{m}$ and estimated values $w_{m}$ are shown in panels c and d, averaged over 100 replicates and blocks of 10 learning trials. As seen in Figure 1d, flexible and constant learning rates show similar performance in the first phase of learning, with only a slight advantage for flexible rates in achieving better estimates of the true values.

**Figure 2:**
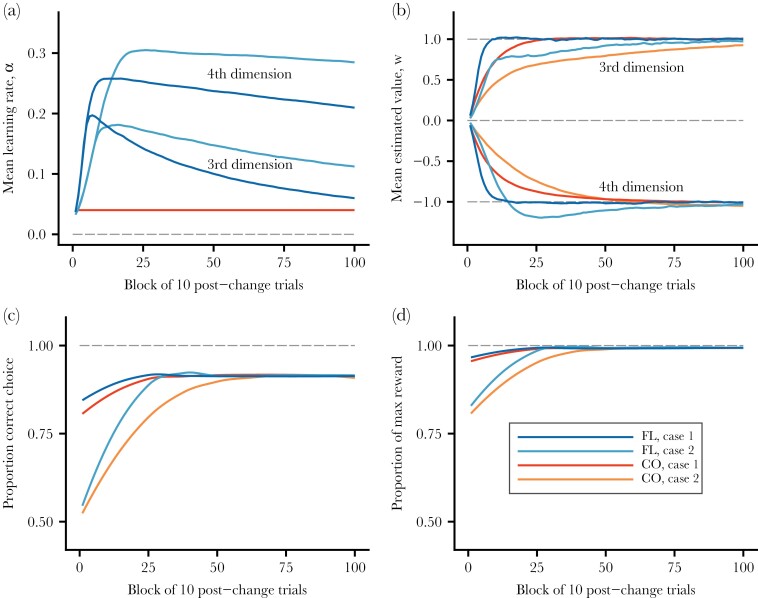
Comparisons for the second phase of learning, when the world becomes more complex, between flexible (FL) and constant (CO) learning rates, and for the two cases studied. Color coding in panel (d) applies to all panels. (a) Average learning rates for the different learning rules and cases. As an illustration, the third and fourth stimulus dimensions are shown. Note that the features in these dimensions were not present in the first phase. The results are similar for the other new dimensions in Table 1 (dimensions 5–10 for case 2). (b) Average estimated values for the different learning rules and cases, for stimulus dimensions 3 and 4. (c) Proportion of choices that are correct, in the sense of the individual choosing the compound stimulus with higher true value. (d) Proportion of reward gained out of the maximum true expected reward available in a trial.

**Figure 3:**
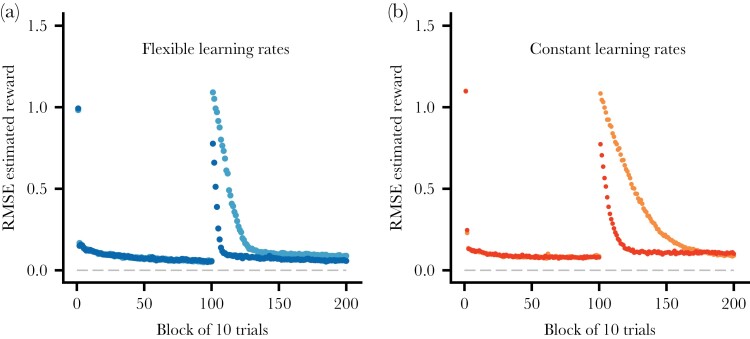
Illustration of the root mean square error (RMSE) of the individual’s estimate (Q) of the reward from the selected compound stimulus, plotted against the trial block, over both phases of learning. There are 10 trials in a block and data are averages over 100 replicate learning simulations. (RMSE is similar to a standard deviation but instead measuring the deviation of an estimate from the true value.) (a) Flexible learning rates. (b) Constant learning rates (α=0.04). The color coding is as in Figure 2d.

Cases 1 and 2 differ in the second phase of learning, having 4 and 10 compound stimuli to discriminate, and the outcome of the learning simulations is illustrated in Figure 2. The flexible learning rates increase sharply in the second phase (Figure 2a), especially for case 2, where many new stimulus dimensions are needed for discrimination. Another comparison of the performance of flexible and constant rates for the two simulated cases appears in Figure 3. In this figure, the performance is measured in terms of the deviation of an individual’s estimate from the true value, implemented as the root mean square error (RMSE). Flexible rates are noticeably better than constant rates in reducing the errors in the value estimates but, as seen from the Figures 1 and 2, RMSE is not the only thing that matters. Thus, even if a learner deviates in its estimates, it can still be the case that it makes a correct choice between two compound stimuli because the deviations might be similar for the two stimuli. Quantitatively, over the first 250 post-change trials for case 2, flexible rates have a loss of 7.5% of the maximum possible reward per trial, whereas constant rates have a higher loss of 11.0%. Over the first 500 post-change trials, these losses are 4.1% and 6.6%. Thus, flexible rates are better than constant rates in handling a transition to a more complex world, but the differences are moderate, and not dramatic, seen over timescales of several hundreds of trials.

In the supplements, we analyze cases similar to those in Figures 1–3, but with high stochasticity (Supplementary Figures S1–S3). Compared to the cases with lower stochasticity, the flexible learning rates become lower, resulting in better estimates of the true values (Supplementary Figure S3), but there are no dramatic additional advantages for flexible over constant rates in gaining rewards (Supplementary Figure S2). Extending the phases of learning to *T* = 10,000 trials (Supplementary Figures S4–S6), the lowering of the flexible learning rates is even more pronounced, in particular in the first phase of learning (Supplementary Figure S4). As a result, flexible rates do much better than constant rates in estimating the true values (Supplementary Figure S6).

A different type of analysis of transitions to a more complex world is to consider how much an individual who fails to learn anything new about the more complex world would lose in terms of rewards. For our cases 1 and 2, this would mean that individuals base their choices only on compound stimulus size, also after the transition. Quantitatively, for case 1, where the new world is only moderately more complex, using only size to choose in the second phase would result in a reward loss of around 7.4% per trial, and the corresponding figure for case 2 is around 22% per trial, which is a substantial loss. Note that these losses would apply to all 1000 trials in the second phase and would be approximately the same for (appropriately modified versions of) our implementations of flexible and constant learning rates. It follows that fairly large advantages can be gained by learning about the new stimulus dimensions in the more complex world.

### Reversal learning

**Figure 4:**
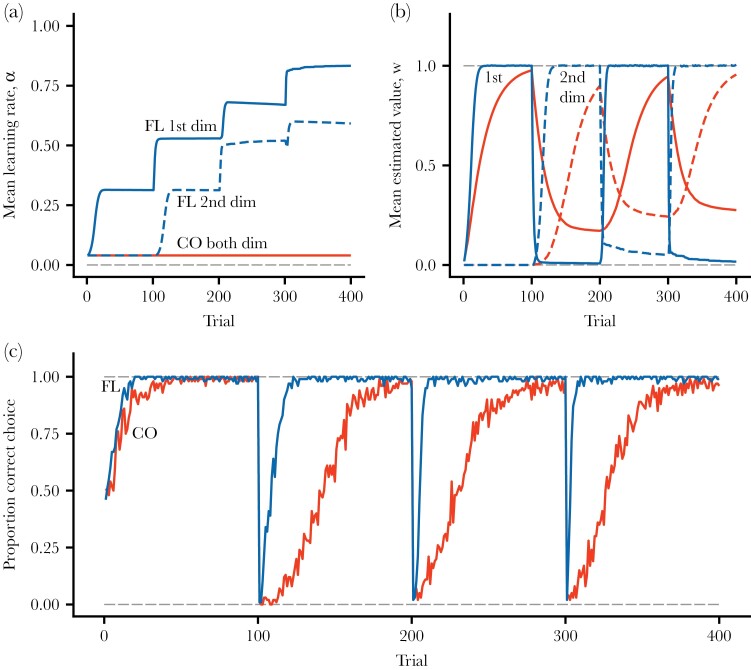
Reversal learning simulation. There are two stimulus dimensions, with 0/1 features that each indicate a type of stimulus. In the first phase of 100 trials, choosing the stimulus with a feature in dimension 1 is rewarded (R=1) and choosing the other, with a feature in dimension 2, is not rewarded (R=0). In the next 100 trials, the rewards are reversed, and then the procedure is repeated for another 200 trials. (a) Average of flexible (FL) and constant (CO) learning rates. (b) Average estimated values for flexible and constant rates, for the two stimulus dimensions. (c) Proportion of choices that are correct, in the sense of the individual choosing the stimulus with higher true value.

A comparison of the performance of flexible and constant rates in reversal learning appears in Figure 4. The flexible rates increase sharply with each successive change in rewards (Figure 4a), but this cannot happen with constant rates. As a consequence, with flexible rates the performance increases over successive reversals (Figure 4b,c), and the increased performance is present already for the first reversal. This means that learning-rate flexibility, for instance, as implemented here, could contribute to observed increases in performance over successive episodes of reversal learning.

### Learning set formation

**Figure 5:**
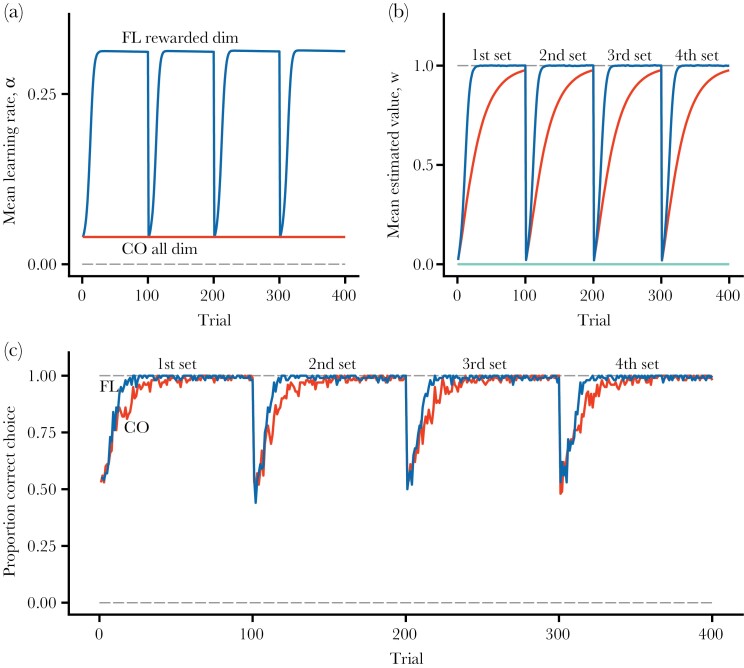
Learning set formation. There are 8 stimulus dimensions, with 0/1 features that each indicate a type of stimulus. In the first phase of 100 trials, there is a choice between the first pair of stimuli, where one is rewarded (R=1) and the other is not rewarded (R=0). In the next 100 trials, a new pair of rewarded and non-rewarded stimuli is used, and so on until four pairs have been used. (a) Average of flexible (FL) and constant (CO) learning rates. (b) Average estimated values for the rewarded stimulus dimension in each set, and for the non-rewarded dimension (green curve at bottom). (c) Proportion of choices that are correct, in the sense of the individual choosing the stimulus with higher true value.

Figure 5 shows a similar comparison of the performance in learning set formation. In this case, there is no additional increase in flexible learning rates over successive shifts in pairs of stimuli (Figure 5a), and consequently no increase in performance (Figure 5b,c). Thus, in contrast to reversal learning, learning-rate flexibility does not increase the performance in learning set formation over successive shifts in stimuli to discriminate.

## DISCUSSION

In our comparisons of flexible (Autostep) and constant (Rescorla-Wagner) learning rates, we found pronounced variation in the flexible learning rates (Figures 1c, 2a, 4a, 5a). As a consequence, flexible rates performed better than constant rates in estimating the true values of different stimulus dimensions (Figures 1d, 2b, 3, 4b, 5b). For our simulated cases of transitions from a simpler to a more complex world, this meant that there were more correct choices (Figure 2c) and higher post-transition rewards (Figure 2d) with flexible than with constant rates. The effects of flexible rates on rewards were moderate but might still be large enough for this kind of learning flexibility to evolve. Alternatively, flexible learning rates could be an aspect of broader cognitive adaptations, relating to attention, memory, and the handling of environmental and social complexity (Emery and Clayton 2004; Deaner et al. 2006; Bond et al. 2007; Izquierdo et al. 2017; Rmus et al. 2021; Leimar et al. 2022).

One can gain an intuitive understanding of flexible learning from the parallel changes in learning rates and estimated values in our results. A learning rate increases as long as there is consistent change, either increase or decrease, in the corresponding estimated value, as illustrated by the blues curves in Figure 2a,b. These curves are averages over block of trials and replicates, and the individual’s learning rule (Autostep) detects such statistical trends in changes in estimated values and adjusts the learning rates. The possible neural implementation of such a mechanism is not known, but it need not fall into the category of higher cognition.

Our analysis of two frequently used measures of behavioral flexibility produced contrasting results. We found that flexible rates resulted in increased performance in reversal learning with each switch in rewards (Figure 4c). This means that a mechanism similar to the one causing the learning rates of our implementation (Autostep) to increase—involving sensitivity to prediction errors that consistently change estimated values over several trials—might contribute to observed improvements in performance in reversal learning with successive switches. In contrast, we found no similar increase in the performance for flexible rates over successive shifts of stimuli in learning set formation (Figure 5c). The reason is that, when there is an entirely new situation, in the sense that all stimulus components that a learner encounters come from new stimulus dimensions, the learning rates for our implementation (Autostep) for these dimensions start from scratch. The same ought to hold for other learning mechanisms that do not increase learning rates for stimulus dimensions that a learner so far has not encountered. A tentative conclusion is that increasing performance in reversal learning and in learning set formation correspond to distinct cognitive capacities, in agreement with the prevailing view in neuroscience (Logue and Gould 2014).

The possibility of different learning rates for different stimulus dimensions is an important aspect of the Rescorla-Wagner model, which we used to implement constant learning rates. The original aim of the model was to explain phenomena such as overshadowing and blocking (Rescorla and Wagner 1972; Miller et al. 1995), and overshadowing of one stimulus component by another depends on differences in learning rates. This is often described in terms of the salience or associability of stimulus components. In nature, the perceived salience of different stimulus components might be adaptive for a particular group of animals. For instance, for some birds, the color of artificial prey is more salient than the shape (Kazemi et al. 2014), and such higher learning rates for color might be adaptive. It is learning rate constancy over time, not over stimulus dimensions, that holds for the Rescorla-Wagner model. Our assumption of the same Rescorla-Wagner learning rate for different stimulus dimensions is thus not at all necessary but is used as a convenient default in the comparison with Autostep.

### Learning models

Many learning models have been proposed in the literature, apart from the ones we study here. Some were developed by experimental psychologists and focus on classical conditioning (Mackintosh 1975; Pearce and Hall 1980; Le Pelley 2010; Pearce and Mackintosh 2010; Esber and Haselgrove 2011). Although these approaches contain interesting and influential ideas, they turn out not to be suitable for our learning simulations here. The reason is that the specific algorithms have difficulties handling large numbers of stimulus dimension and, furthermore, only allow for fairly limited variation in learning rates.

These approaches discuss variation in learning rates in terms of the effects of attention on learning. The idea that attention to stimulus components could be important for learning is often put forward and has been investigated experimentally (Beesley et al. 2015; Niv et al. 2015; Leong et al. 2017; Torrents-Rodas et al. 2021). Nevertheless, models with variation learning rates need not explicitly include attention as a mechanism (Dayan et al. 2000), and Autostep is an example of this.

There are also Kalman-filter-inspired learning models (Sutton 1992b; Dayan et al. 2000; Gershman 2015). The Kalman filter originated in the engineering-related fields of optimization and control and gives an optimal solution to a control problem in certain mathematically well-defined situations. It can be used to construct optimal learning algorithms in certain cases where the relative magnitudes of volatility and stochasticity are known (Dayan et al. 2000; Gershman 2015; Piray and Daw 2021). In many situations where the Kalman filter is optimal, the IDBD algorithm achieves approximately the same performance (Sutton 1992b). Because the Autostep algorithm is similar to IDBD, it is reasonable to expect that it has approximately the same performance as a Kalman filter model in situations where the Kalman filter is optimal. A seeming advantage for algorithms such as Autostep and IDBD over a Kalman filter is that they do not require a priori knowledge of the relative magnitudes of volatility and stochasticity.

There is much work in theoretical neuroscience on neural-network-based learning models. This work is of interest if it helps in identifying neural correlates of learning phenomena. An influential example is the modeling by Wang et al. (2018). They present a general perspective on meta-learning and report on learning simulations for situations similar to reversal learning and learning set formation. In one simulation, they trained a network to obtain rewards in situations with changing volatility, and the network then showed higher learning rates for higher reward volatility, in a similar way as was found in an experiment by Behrens et al. (2007). In another simulation, a network was trained on learning set formation and subsequently showed increasing performance similar to what was found in the original experiments by Harlow (1949). These are interesting results, but it is not clear which kinds of cognitive mechanisms caused the networks to succeed in the learning tasks.

### Behavioral flexibility

The idea that behavioral flexibility should be adaptive in complex worlds is well established. There is evidence that animals that are known or believed to have the cognitive capacities associated with a larger brain, and thus presumably show more flexible behavior, are more successful in novel environments (Sol et al. 2002, 2005). Conversely, there is evidence suggesting that invasive species have cognitive abilities that allow flexible behavior (Szabo et al. 2020). Among the many examples of ecologically relevant situations where there can be a shift to a more complex world are invasions into new habitats (Vardi and Berger-Tal 2022) but also new contextual cues for food choice (Hansen et al. 2010). In nature, individuals are likely to experience environmental changes of many kinds, including the introduction of new significant stimulus dimensions and changes, and even reversals, in the information content of previously encountered stimulus dimensions.

For learning-rate flexibility, which is the focus of our investigation here, it is worth noting that flexibility does not only entail higher learning rates for higher reward volatility, but also lower learning rates for higher reward stochasticity (as seen by comparing Figures 1 and 2 with Supplementary Figures S1, S2, S4, and S5). While the effects of reward stochasticity have been investigated in neuroscience (Nassar et al. 2010; Piray and Daw 2021), there seems to be a lack of studies on the ecological relevance of low versus high learning rates.

Reversal learning experiments, in particular those involving serial reversals, can detect increasing learning rates with repeated reward volatility, as illustrated by Figure 4. One focus of such studies has been whether species or groups of species differ in this performance. For instance, Bond et al. (2007) compared three corvid species, each of which showed increasing performance with successive reversals, but to different degrees, and suggested that differences in social complexity could explain the observation. Another example is that, based on overviews of several studies, there appears to be a pattern of little or no increase in performance over successive reversals in species of fish (Bitterman 1975; Boussard et al. 2020), in contrast to what is found in other groups of vertebrates. There is so far no well-established explanation for this possible difference. In our learning simulations of transitions to a more complex world, we used cleaner fish as an illustrative example, but up to now, there are no serial reversal learning experiments on these, and it is not known to what extent they show flexible learning rates. Thus, in principle, cleaner fish learning might be better described by the Rescorla-Wagner model, for instance, with adaptive but not very flexible learning rates for different stimulus dimensions, than by the Autostep model. Additional experiments are needed to settle the issue.

A number of studies have examined the neural correlates of learning rate flexibility. One general conclusion is that for humans and non-human primates, as well as for rodents, regions in the prefrontal cortex are important for reversal learning (Izquierdo et al. 2017), with serotonin neurons playing a role (Grossman et al. 2022). In fish, learning experiments on selection lines have shown that brain size influences performance in reversal learning (Buechel et al. 2018) and its decline with age (Boussard et al. 2021), and that specifically relative telencephalon size influences the performance in reversal learning (Triki et al. 2022, 2023). In general, investment in brain tissue could represent a cost of cognitive flexibility.

Our simulations showed a qualitative difference between performance in serial reversal learning (Figure 4) and in learning set formation (Figure 5), consistent with the idea that these depend on distinct cognitive capabilities. The capacity for learning set formation appears to have a more narrow phylogenetic distribution, being largely restricted to primates (Harlow 1949; Warren 1966; Deaner et al. 2006) and some species of birds (Wilson et al. 1985; Emery and Clayton 2004; de Mendonça-Furtado and Ottoni 2008), than has increasing performance in serial reversal learning. Learning set formation is sometimes described as rule learning, with the rule being “win-stay, lose-shift” (Warren 1966; Mackintosh et al. 1968; Emery and Clayton 2004), but the actual cognitive mechanism involved is not known. Furthermore, seemingly abstract rules that an experimenter has imposed (e.g., “precisely one out of two possibilities is rewarded”) need not correspond to important situations encountered in nature. It might be more important for animals to learn rules about categories of compound stimuli, for instance “predator” and “non-predator.” Cleaner fish appear to use this categorization to solve a problem of “avoiding punishment” (Wismer et al. 2016).

Overall, our simulations show that adaptive learning rate flexibility can rely on relatively simple mechanisms, such as using correlations between current and recent changes in estimated values to adjust rates, as for Autostep. To the extent that our examples of transitions to a complex world are biologically realistic, one can also conclude that learning rate flexibility gives a clear but only moderately large advantage over constant rates. In comparison, as we have shown, it is considerably more important to learn at all about the new and informative stimulus dimensions in the complex world. Cognitive capacities allowing individuals to achieve this seem essential for behavioral flexibility and might involve attention, memory, and exploration, in addition to flexible learning rates.

## Supplementary Material

arad109_suppl_Supplementary_Figures_S1-S6

arad109_suppl_Supplementary_Figures_S1-S6

## Data Availability

Analyses reported in this article can be reproduced using the code provided by Leimar (2023).

## References

[CIT0001] Audet J-N , Lefebvre L. 2017. What’s flexible in behavioral flexibility? Behav Ecol. 28(4):943–947. doi:10.1093/beheco/arx007

[CIT0002] Bailey AM , McDaniel WF, Thomas RK. 2007. Approaches to the study of higher cognitive functions related to creativity in nonhuman animals. Methods. 42(1):3–11. doi:10.1016/j.ymeth.2006.12.003 17434410

[CIT0003] Bairlein F , Simons D. 1995. Nutritional adaptations in migrating birds. Isr J Ecol Evol. 41(3):357–367. doi:10.1080/00212210.1995.10688805

[CIT0004] Beesley T , Nguyen KP, Pearson D, Le Pelley ME. 2015. Uncertainty and predictiveness determine attention to cues during human associative learning. Q J Exp Psychol (2006). 68(11):2175–2199. doi:10.1080/17470218.2015.1009919 25832459

[CIT0005] Behrens TEJ , Woolrich MW, Walton ME, Rushworth MFS. 2007. Learning the value of information in an uncertain world. Nat Neurosci. 10(9):1214–1221. doi:10.1038/nn1954 17676057

[CIT0006] Binning SA , Rey O, Wismer S, Triki Z, Glauser G, Soares MC, Bshary R. 2017. Reputation management promotes strategic adjustment of service quality in cleaner wrasse. Sci Rep. 7(1):8425. doi:10.1038/s41598-017-07128-5 28827634 PMC5566447

[CIT0007] Birrell JM , Brown VJ. 2000. Medial frontal cortex mediates perceptual attentional set shifting in the rat. J Neurosci. 20(11):4320–4324. doi:10.1523/JNEUROSCI.20-11-04320.2000 10818167 PMC6772641

[CIT0008] Bitterman ME. 1975. The comparative analysis of learning: are the laws of learning the same in all animals? Science. 188(4189):699–709. 17755167 10.1126/science.188.4189.699

[CIT0009] Bond AB , Kamil AC, Balda RP. 2007. Serial reversal learning and the evolution of behavioral flexibility in three species of North American corvids (*Gymnorhinus cyanocephalus*, *Nucifraga columbiana*, *Aphelocoma californica*). J Comp Psychol. 121(4):372–379. doi:10.1037/0735-7036.121.4.372 18085920

[CIT0010] Boussard A , Amcoff M, Buechel SD, Kotrschal A, Kolm N. 2021. The link between relative brain size and cognitive ageing in female guppies (*Poecilia reticulata*) artificially selected for variation in brain size. Exp Gerontol. 146:111218. doi:10.1016/j.exger.2020.111218 33373711

[CIT0011] Boussard A , Buechel SD, Amcoff M, Kotrschal A, Kolm N. 2020. Brain size does not predict learning strategies in a serial reversal learning test. J Exp Biol. 223(15):jeb224741. doi:10.1242/jeb.224741 32561630 PMC7413604

[CIT0012] Bshary R. 2001. The cleaner fish market. In: Noë R, Van Hooff JARAM, Hammerstein P, editors. Economics in nature: social dilemmas, mate choice and biological markets. Cambridge (UK): Cambridge University Press. p. 146–172. doi:10.1017/CBO9780511752421.010

[CIT0013] Bshary R , Grutter AS. 2002. Asymmetric cheating opportunities and partner control in a cleaner fish mutualism. Anim Behav. 63(3):547–555. doi:10.1006/anbe.2001.1937

[CIT0014] Buechel SD , Boussard A, Kotrschal A, van der Bijl W, Kolm N. 2018. Brain size affects performance in a reversal-learning test. Proc Biol Sci. 285(1871):20172031. doi:10.1098/rspb.2017.2031 29367391 PMC5805926

[CIT0015] Cauchoix M , Hermer E, Chaine AS, Morand-Ferron J. 2017. Cognition in the field: comparison of reversal learning performance in captive and wild passerines. Sci Rep. 7(1):12945. doi:10.1038/s41598-017-13179-5 29021558 PMC5636806

[CIT0016] Côté IM. 2000. Evolution and ecology of cleaning symbioses in the sea. In: Gibson RN, Barnes M, editors. Oceanography and marine biology. Vol. 38. New York (NY): Taylor & Francis. p. 311–355.

[CIT0017] Dayan P , Kakade S, Montague PR. 2000. Learning and selective attention. Nat Neurosci. 3(11):1218–1223. doi:10.1038/81504 11127841

[CIT0018] de Mendonça-Furtado O , Ottoni EB. 2008. Learning generalization in problem solving by a blue-fronted parrot (*Amazona aestiva*). Anim Cogn. 11(4):719–725. doi:10.1007/s10071-008-0168-x 18575906

[CIT0019] Deaner RO , van Schaik CP, Johnson V. 2006. Do some taxa have better domain-general cognition than others? A meta-analysis of nonhuman primate studies. Evol Psychol. 4(1):147470490600400114. doi:10.1177/147470490600400114

[CIT0020] Diederen KMJ , Schultz W. 2015. Scaling prediction errors to reward variability benefits error-driven learning in humans. J Neurophysiol. 114(3):1628–1640. doi:10.1152/jn.00483.2015 26180123 PMC4563025

[CIT0021] Emery NJ , Clayton NS. 2004. The mentality of crows: convergent evolution of intelligence in corvids and apes. Science. 306(5703):1903–1907. doi:10.1126/science.1098410 15591194

[CIT0022] Esber GR , Haselgrove M. 2011. Reconciling the influence of predictiveness and uncertainty on stimulus salience: a model of attention in associative learning. Proc Biol Sci. 278(1718):2553–2561. doi:10.1098/rspb.2011.0836 21653585 PMC3136838

[CIT0023] Farashahi S , Xu J, Wu S-W, Soltani A. 2020. Learning arbitrary stimulus-reward associations for naturalistic stimuli involves transition from learning about features to learning about objects. Cognition. 205:104425. doi:10.1016/j.cognition.2020.104425 32958287

[CIT0024] Fawcett TW , Hamblin S, Giraldeau LA. 2013. Exposing the behavioral gambit: the evolution of learning and decision rules. Behav Ecol. 24(1):2–11. doi:10.1093/beheco/ars085

[CIT0025] Gershman SJ. 2015. A unifying probabilistic view of associative learning. PLoS Comput Biol. 11(11):e1004567. doi:10.1371/journal.pcbi.1004567 26535896 PMC4633133

[CIT0026] Grossman CD , Bari BA, Cohen JY. 2022. Serotonin neurons modulate learning rate through uncertainty. Curr Biol. 32(3):586–599.e7. doi:10.1016/j.cub.2021.12.006 34936883 PMC8825708

[CIT0027] Grutter AS. 1994. Spatial and temporal variations of the ectoparasites of seven reef fish species from Lizard Island and Heron Island, Australia. Mar Ecol Prog Ser. 115:21–30. doi:10.3354/meps115021

[CIT0028] Grutter AS. 1995. Relationship between cleaning rates and ectoparasite loads in coral reef fishes. Mar Ecol Prog Ser. 118:51–58. doi:10.3354/meps118051

[CIT0029] Grutter AS , Bshary R. 2003. Cleaner wrasse prefer client mucus: support for partner control mechanisms in cleaning interactions. Proc Biol Sci. 270(suppl_2):S242–S244. doi:10.1098/rsbl.2003.0077 14667394 PMC1809949

[CIT0030] Grutter AS , Bshary R. 2004. Cleaner fish, *Labroides dimidiatus*, diet preferences for different types of mucus and parasitic gnathiid isopods. Anim Behav. 68(3):583–588. doi:10.1016/j.anbehav.2003.11.014

[CIT0031] Hansen BT , Holen OH, Mappes J. 2010. Predators use environmental cues to discriminate between prey. Behav Ecol Sociobiol. 64(12):1991–1997. doi:10.1007/s00265-010-1010-4

[CIT0032] Harlow HF. 1949. The formation of learning sets. Psychol Rev. 56(1):51–65. doi:10.1037/h0062474 18124807

[CIT0033] Holland PC , Schiffino FL. 2016. Mini-review: prediction errors, attention and associative learning. Neurobiol Learn Mem. 131:207–215. doi:10.1016/j.nlm.2016.02.014 26948122 PMC4862921

[CIT0034] Izquierdo A , Brigman JL, Radke AK, Rudebeck PH, Holmes A. 2017. The neural basis of reversal learning: an updated perspective. Neuroscience. 345:12–26. doi:10.1016/j.neuroscience.2016.03.021 26979052 PMC5018909

[CIT0035] Jacobs RA. 1988. Increased rates of convergence through learning rate adaptation. Neural Netw. 1(4):295–307. doi:10.1016/0893-6080(88)90003-2

[CIT0036] Janmaat KRL , Boesch C, Byrne R, Chapman CA, Goné Bi ZB, Head JS, Robbins MM, Wrangham RW, Polansky L. 2016. Spatio-temporal complexity of chimpanzee food: How cognitive adaptations can counteract the ephemeral nature of ripe fruit. Am J Primatol. 78(6):626–645. doi:10.1002/ajp.22527 26800493

[CIT0037] Kazemi B , Gamberale-Stille G, Tullberg BS, Leimar O. 2014. Stimulus salience as an explanation for imperfect mimicry. Curr Biol. 24(9):965–969. doi:10.1016/j.cub.2014.02.061 24726157

[CIT0038] Le Pelley ME. 2010. The hybrid modeling approach to conditioning. In: Schmajuk N, editor. Computational models of conditioning. Cambridge (UK): Cambridge University Press. p. 71–107. doi:10.1017/CBO9780511760402.004

[CIT0039] Lea SEG , Chow PKY, Leaver LA, McLaren IPL. 2020. Behavioral flexibility: a review, a model, and some exploratory tests. Learn Behav. 48(1):173–187. doi:10.3758/s13420-020-00421-w 32043268 PMC7082303

[CIT0040] Leimar O. 2023. Code for: Flexible learning in complex worlds. Behav Ecol. doi:10.5281/zenodo.10170754. https://zenodo.org/doi/10.5281/zenodo.10170754 PMC10756056 38162692

[CIT0041] Leimar O , Dall SRX, Houston AI, McNamara JM. 2022. Behavioural specialization and learning in social networks. Proc Biol Sci. 289(1980):20220954. doi:10.1098/rspb.2022.0954 35946152 PMC9363987

[CIT0042] Leong YC , Radulescu A, Daniel R, DeWoskin V, Niv Y. 2017. Dynamic interaction between reinforcement learning and attention in multidimensional environments. Neuron. 93(2):451–463. doi:10.1016/j.neuron.2016.12.040 28103483 PMC5287409

[CIT0043] Liu Y , Day LB, Summers K, Burmeister SS. 2016. Learning to learn: advanced behavioural flexibility in a poison frog. Anim Behav. 111:167–172. doi:10.1016/j.anbehav.2015.10.018

[CIT0044] Logue SF , Gould TJ. 2014. The neural and genetic basis of executive function: attention, cognitive flexibility, and response inhibition. Pharmacol Biochem Behav. 123:45–54. doi:10.1016/j.pbb.2013.08.007 23978501 PMC3933483

[CIT0045] Mackintosh NJ. 1975. A theory of attention: variations in the associability of stimuli with reinforcement. Psychol Rev. 82(4):276–298. doi:10.1037/h0076778

[CIT0046] Mackintosh NJ , Mcgonigle B, Holgate V, Vanderver V. 1968. Factors underlying improvement in serial reversal learning. Can J Psychol. 22(2):85–95. doi:10.1037/h0082753 5649040

[CIT0047] Mahmood AR , Sutton RS, Degris T, Pilarski PM. 2012. Tuning-free step-size adaptation. In: 2012 IEEE International Conference on Acoustics, Speech and Signal Processing (ICASSP). p. 2121–2124. https://ieeexplore.ieee.org/abstract/document/6288330

[CIT0048] Miller RR , Barnet RC, Grahame NJ. 1995. Assessment of the Rescorla-Wagner model. Psychol Bull. 117(3):363–386. doi:10.1037/0033-2909.117.3.363 7777644

[CIT0049] Nassar MR , Wilson RC, Heasly B, Gold JI. 2010. An approximately Bayesian delta-rule model explains the dynamics of belief updating in a changing environment. J Neurosci. 30(37):12366–12378. doi:10.1523/JNEUROSCI.0822-10.2010 20844132 PMC2945906

[CIT0050] Niv Y , Daniel R, Geana A, Gershman SJ, Leong YC, Radulescu A, Wilson RC. 2015. Reinforcement learning in multidimensional environments relies on attention mechanisms. J Neurosci. 35(21):8145–8157. doi:10.1523/JNEUROSCI.2978-14.2015 26019331 PMC4444538

[CIT0051] Pearce JM , Hall G. 1980. A model for Pavlovian learning: variations in the effectiveness of conditioned but not of unconditioned stimuli. Psychol Rev. 87(6):532–552. doi:10.1037/0033-295X.87.6.532 7443916

[CIT0052] Pearce JM , Mackintosh NJ. 2010. Two theories of attention: a review and a possible integration. In: Mitchell C, Le Pelley ME, editors. Attention and associative learning: from brain to behaviour. Oxford: Oxford University Press. p. 11–39; https://orca.cardiff.ac.uk/id/eprint/31042/

[CIT0053] Pierce BJ , McWilliams SR. 2005. Seasonal changes in composition of lipid stores in migratory birds: causes and consequences. The Condor. 107(2):269–279. doi:10.1093/condor/107.2.269

[CIT0054] Piray P , Daw ND. 2021. A model for learning based on the joint estimation of stochasticity and volatility. Nat Commun. 12(1):6587. doi:10.1038/s41467-021-26731-9 34782597 PMC8592992

[CIT0055] Raine NE , Chittka L. 2012. No trade-off between learning speed and associative flexibility in bumblebees: a reversal learning test with multiple colonies. PLoS One. 7(9):e45096. doi:10.1371/journal.pone.0045096 23028779 PMC3447877

[CIT0056] Rescorla RA , Wagner AR. 1972. A theory of Pavlovian conditioning: variations in the effectiveness of reinforcement and nonreinforcement. In: Black AH, Prokasy WF, editors. Classical conditioning II: current research and theory. New York: Appleton-Century-Crofts. p. 64–99.

[CIT0057] Rmus M , McDougle SD, Collins AG. 2021. The role of executive function in shaping reinforcement learning. Curr Opin Behav Sci. 38:66–73. doi:10.1016/j.cobeha.2020.10.003 35194556 PMC8859995

[CIT0058] Roche DG , Jornod M, Douet V, Grutter AS, Bshary R. 2021. Client fish traits underlying variation in service quality in a marine cleaning mutualism. Anim Behav. 175:137–151. doi:10.1016/j.anbehav.2021.03.005

[CIT0059] Sol D , Duncan RP, Blackburn TM, Cassey P, Lefebvre L. 2005. Big brains, enhanced cognition, and response of birds to novel environments. Proc Natl Acad Sci USA. 102(15):5460–5465. doi:10.1073/pnas.0408145102 15784743 PMC556234

[CIT0060] Sol D , Timmermans S, Lefebvre L. 2002. Behavioural flexibility and invasion success in birds. Anim Behav. 63(3):495–502. doi:10.1006/anbe.2001.1953

[CIT0061] Soltani A , Izquierdo A. 2019. Adaptive learning under expected and unexpected uncertainty. Nat Rev Neurosci. 20(10):635–644. doi:10.1038/s41583-019-0180-y 31147631 PMC6752962

[CIT0062] Sutton RS. 1992a. Adapting bias by gradient descent: an incremental version of delta-bar-delta. In: Proceedings of the Tenth National Conference on Artificial Intelligence. San Jose, CA: AAAI Press. (AAAI’92). p. 171–176.

[CIT0063] Sutton RS. 1992b. Gain adaptation beats least squares? In: Proceedings of the Seventh Yale Workshop on Adaptive and Learning Systems. New Haven, CT: Yale University. p. 161–166.

[CIT0064] Sutton RS. 2022. A history of meta-gradient: gradient methods for meta-learning. arXiv preprint. http://arxiv.org/abs/2202.09701

[CIT0065] Sutton RS , Barto AG. 2018. Reinforcement learning: an introduction, second edition. Cambridge (MA): MIT Press.

[CIT0066] Szabo B , Damas-Moreira I, Whiting MJ. 2020. Can cognitive ability give invasive species the means to succeed? A review of the evidence. Front Ecol Evol. 8. doi:10.3389/fevo.2020.00187

[CIT0067] Topel S , Ma I, Sleutels J, van Steenbergen H, de Bruijn ERA, van Duijvenvoorde ACK. 2023. Expecting the unexpected: a review of learning under uncertainty across development. Cogn Affect Behav Neurosci. 23(3):718–738. doi:10.3758/s13415-023-01098-0 37237092 PMC10390612

[CIT0068] Torrents-Rodas D , Koenig S, Uengoer M, Lachnit H. 2021. Evidence for two attentional mechanisms during learning. Q J Exp Psychol. 74(12):2112–2123. doi:10.1177/17470218211019308 33957827

[CIT0069] Triki Z , Emery Y, Teles MC, Oliveira RF, Bshary R. 2020. Brain morphology predicts social intelligence in wild cleaner fish. Nat Commun. 11(1):6423. doi:10.1038/s41467-020-20130-2 33349638 PMC7752907

[CIT0070] Triki Z , Fong S, Amcoff M, Vàsquez-Nilsson S, Kolm N. 2023. Experimental expansion of relative telencephalon size improves the main executive function abilities in guppy. PNAS Nexus. 2(6):pgad129. doi:10.1093/pnasnexus/pgad129 37346268 PMC10281379

[CIT0071] Triki Z , Granell-Ruiz M, Fong S, Amcoff M, Kolm N. 2022. Brain morphology correlates of learning and cognitive flexibility in a fish species (*Poecilia reticulata*). Proc Biol Sci. 289(1978):20220844. doi:10.1098/rspb.2022.0844 35858069 PMC9277233

[CIT0072] Triki Z , Wismer S, Rey O, Ann Binning S, Levorato E, Bshary R. 2019. Biological market effects predict cleaner fish strategic sophistication. Behav Ecol. 30(6):1548–1557. doi:10.1093/beheco/arz111

[CIT0073] Trimmer PC , McNamara JM, Houston AI, Marshall JAR. 2012. Does natural selection favour the Rescorla–Wagner rule? J Theor Biol. 302:39–52. doi:10.1016/j.jtbi.2012.02.014 22619751

[CIT0074] Uddin LQ. 2021. Cognitive and behavioural flexibility: neural mechanisms and clinical considerations. Nat Rev Neurosci. 22(3):167–179. doi:10.1038/s41583-021-00428-w 33536614 PMC7856857

[CIT0075] Vardi R , Berger-Tal O. 2022. Environmental variability as a predictor of behavioral flexibility in urban environments. Behav Ecol. 33(3):573–581. doi:10.1093/beheco/arac002

[CIT0076] Wang JX , Kurth-Nelson Z, Kumaran D, Tirumala D, Soyer H, Leibo JZ, Hassabis D, Botvinick M. 2018. Prefrontal cortex as a meta-reinforcement learning system. Nat Neurosci. 21(6):860–868. doi:10.1038/s41593-018-0147-8 29760527

[CIT0077] Warren JM. 1966. Reversal learning and the formation of learning sets by cats and rhesus monkeys. J Comp Physiol Psychol. 61(3):421–428. doi:10.1037/h0023269 4957108

[CIT0078] Wilson B , Mackintosh NJ, Boakes RA. 1985. Transfer of relational rules in matching and oddity learning by pigeons and corvids. Q J Exp Psychol Section B. 37(4b):313–332. doi:10.1080/14640748508401173

[CIT0079] Wismer S , Grutter A, Bshary R. 2016. Generalized rule application in bluestreak cleaner wrasse (*Labroides dimidiatus*): using predator species as social tools to reduce punishment. Anim Cogn. 19(4):769–778. doi:10.1007/s10071-016-0975-4 27016339

[CIT0080] Wismer S , Pinto AI, Triki Z, Grutter AS, Roche DG, Bshary R. 2019. Cue-based decision rules of cleaner fish in a biological market task. Anim Behav. 158:249–260. doi:10.1016/j.anbehav.2019.09.013

