# Supplementary material for: Flexible learning in complex worlds
Source: Behav Ecol. 2023 Dec 29;35(1):arad109. doi: 10.1093/beheco/arad109 (PMC10756056; doi:10.1093/beheco/arad109)
Supplement: arad109_suppl_Supplementary_Figures_S1-S6 [file arad109_suppl_supplementary_figures_s1-s6.pdf]

Supplement for ‘Flexible learning in complex worlds’

Olof Leimar, Andrés E Quiñones, and Redouan Bshary

Content: supplementary figures and model details. Citations refer to the list of references in the main text. Equations are numbered with the prefix S; plain equation numbers refer to equations in the main text.

Supplementary figures

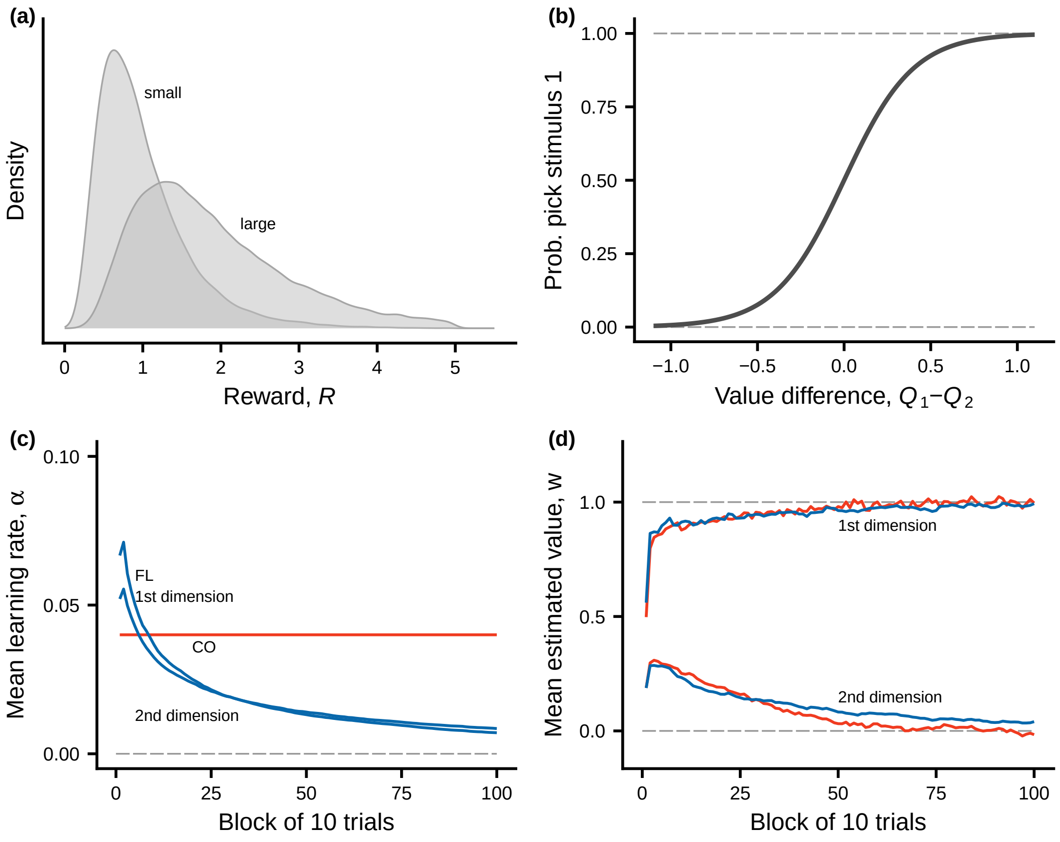

Figure S1: Same as the first cases in the main text (Figure 1), but with high reward stochasticity ($\sigma_R = 0.50$). **(a)** Distribution of rewards from the two types of compound stimuli. **(b)** The function from equation (4), giving the probability of choice from the difference in estimated values of the two compound stimuli present in a trial. **(c)** Average of flexible (FL) and constant (CO) learning rates for the two stimulus dimensions. **(d)** Average estimated values for flexible and constant rates for the two stimulus dimensions (first dimension has true value 1.0 and second has true value 0). There are 10 trials in a block and data are averages over 100 replicate learning simulations.

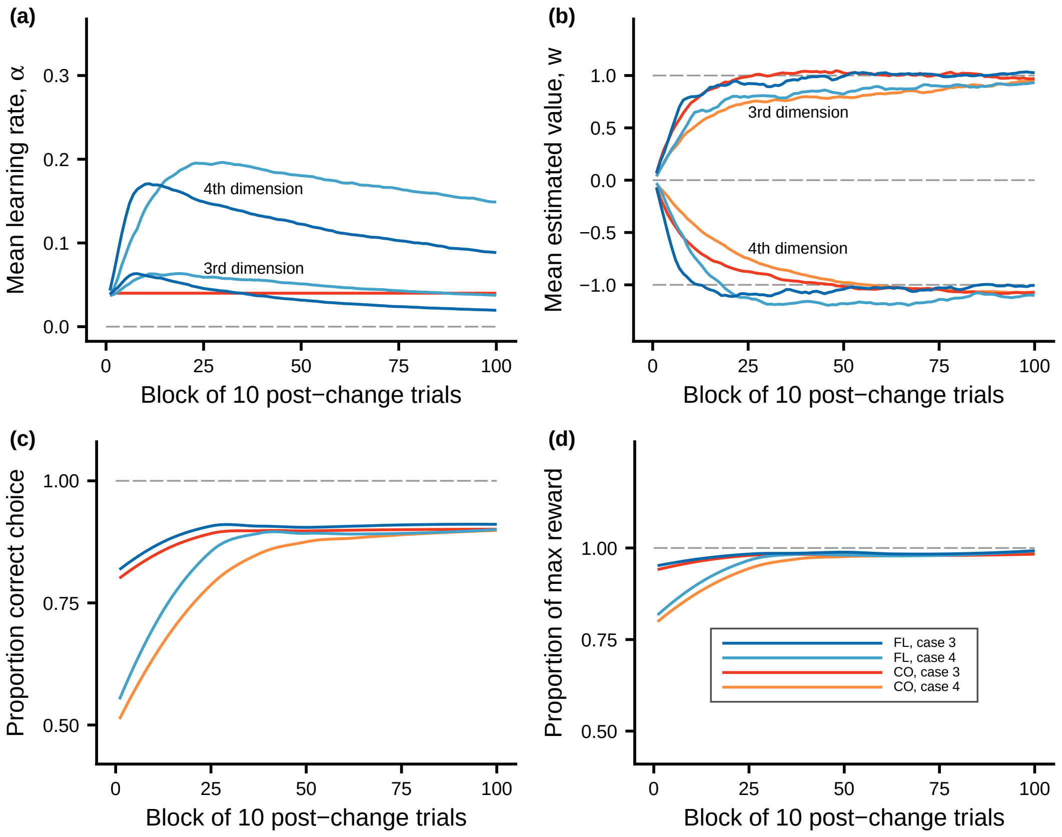

Figure S2: Same as the first cases in the main text (Figure 2), but with high reward stochasticity ($\sigma_R = 0.50$). Comparisons of the second phase of learning, when the world becomes more complex, between flexible (FL) and constant (CO) learning rates, and for the cases studied. Color coding in panel **(d)** applies to all panels. **(a)** Average learning rates for the different learning rules and cases. As an illustration, the third and fourth stimulus dimensions are shown. Note that the features in these dimensions were not present in the first phase. **(b)** Estimated values for the different learning rules and cases, for stimulus dimensions 3 and 4. **(c)** Proportion of choices that are correct, in the sense of the individual choosing the compound stimulus with higher true value. **(d)** Proportion of reward gained out of the maximum true expected reward available in a trial.

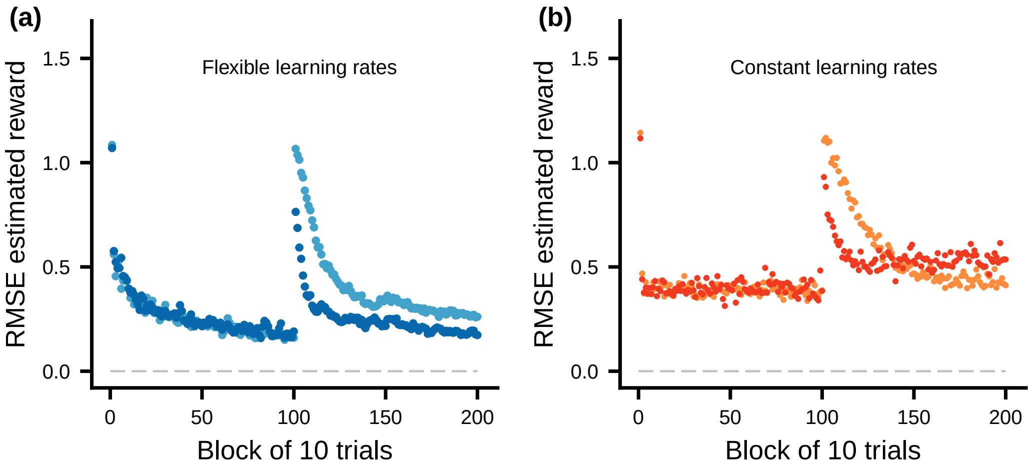

Figure S3: Same as the first cases in the main text (Figure 3), but with high reward stochasticity ($\sigma_R = 0.50$). Illustration of the root mean square error (RMSE) of the individual's estimate (Q) of the reward from the selected compound stimulus, plotted against the trial block, over both phases of learning. There are 10 trials in a block and data are averages over 100 replicate learning simulations. **(a)** Flexible learning rates. **(b)** Constant learning rates ($\alpha = 0.04$). The color coding is as in Figure S2d.

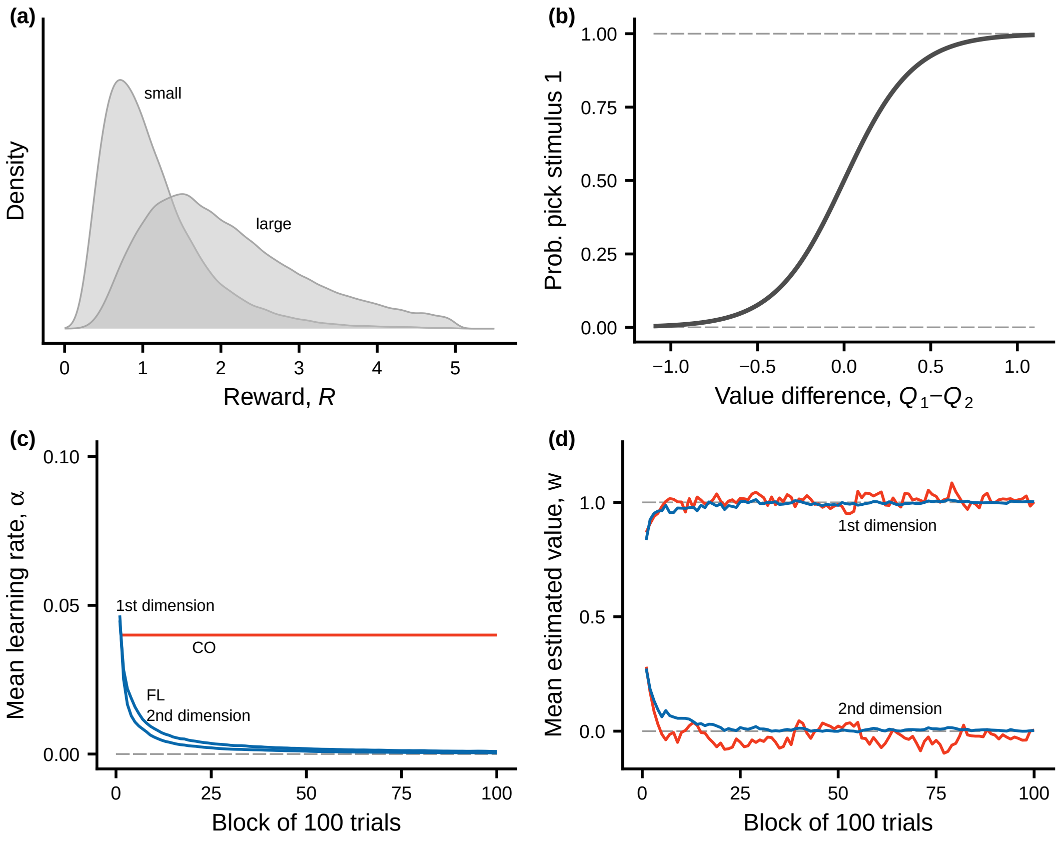

Figure S4: High reward stochasticity ($\sigma_R = 0.50$), as in Figure S1, but the first phase of learning is much longer, with $T = 10000$. **(a)** Distribution of rewards from the two types of compound stimuli. **(b)** The function from equation (4), giving the probability of choice from the difference in estimated values of the two compound stimuli present in a trial. **(c)** Average of flexible (FL) and constant (CO) learning rates for the two stimulus dimensions. **(d)** Average estimated values for flexible and constant rates for the two stimulus dimensions (first dimension has true value 1.0 and second has true value 0). There are 100 trials in a block and data are averages over 10 replicate learning simulations.

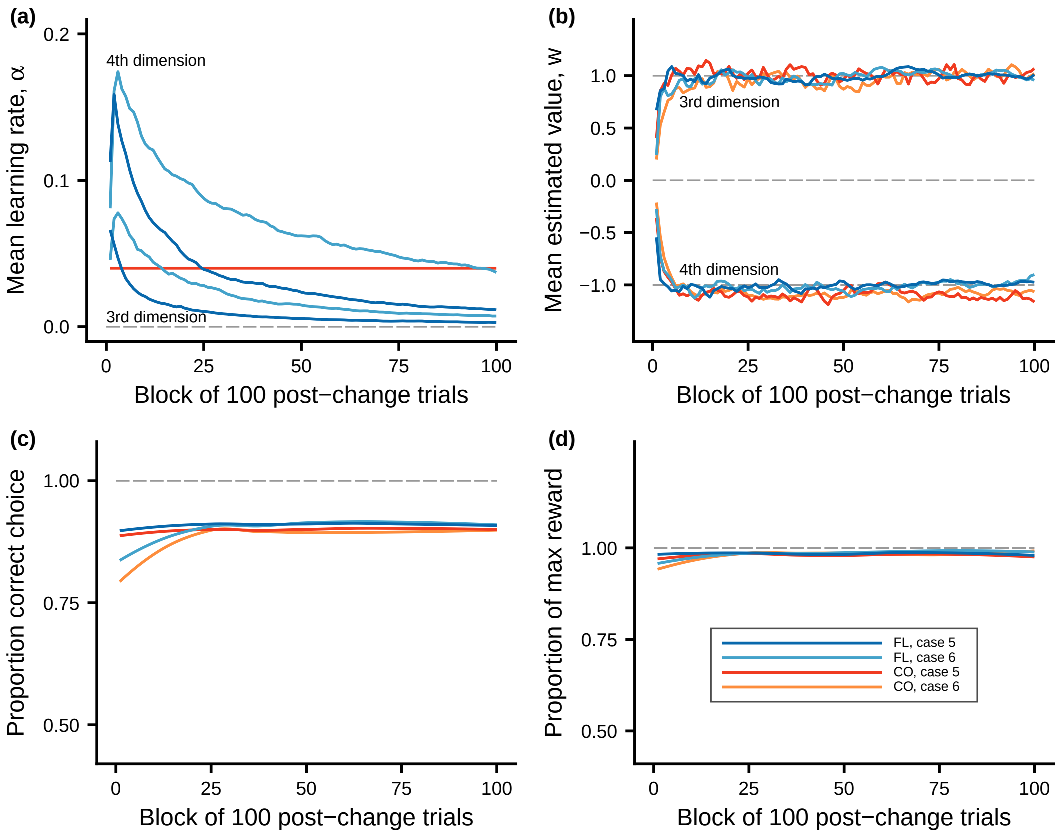

Figure S5: High reward stochasticity ($\sigma_R = 0.50$), as in Figure S2, but the second phase of learning is much longer, with $T = 10000$. Comparisons of the second phase of learning, when the world becomes more complex, between flexible (FL) and constant (CO) learning rates, and for the cases studied. Color coding in panel **(d)** applies to all panels. **(a)** Average learning rates for the different learning rules and cases. As an illustration, the third and fourth stimulus dimensions are shown. Note that the features in these dimensions were not present in the first phase. **(b)** Average estimated values for the different learning rules and cases, for stimulus dimensions 3 and 4. **(c)** Proportion of choices that are correct, in the sense of the individual choosing the compound stimulus with higher true value. **(d)** Proportion of reward gained out of the maximum true expected reward available in a trial.

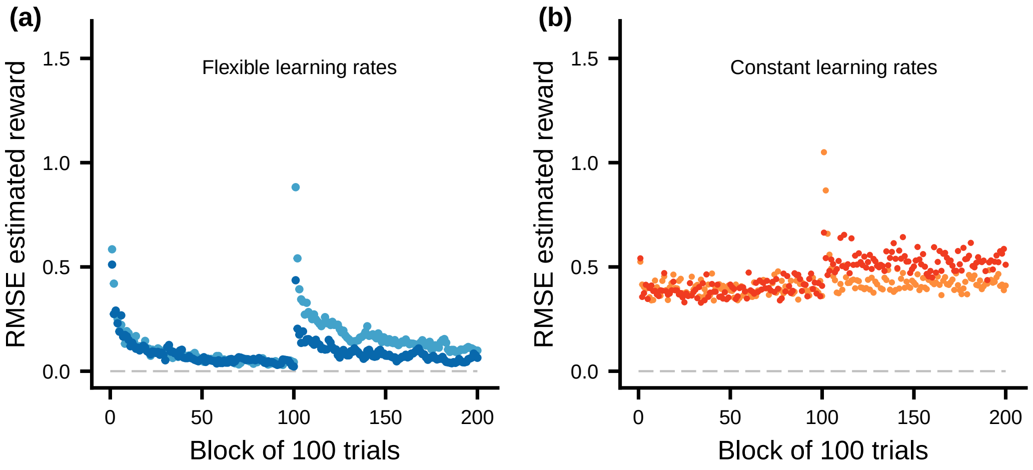

Figure S6: High reward stochasticity ($\sigma_R = 0.50$), as in Figure S3, but the first and second phases of learning are much longer, with $T = 10000$. Illustration of the root mean square error (RMSE) of the individual's estimate (Q) of the reward from the selected compound stimulus, plotted against the trial block, over both phases of learning. There are 100 trials in a block and data are averages over 10 replicate learning simulations. **(a)** Flexible learning rates. **(b)** Constant learning rates ($\alpha = 0.04$). The color coding is as in Figure S5d.

Model details

Using the notation W_m for the true expected value for a stimulus dimension (e.g., as given in Table 1 of the main text), the true expected reward from a compound stimulus with stimulus components or features x_m, $m = 1, \ldots, M$, is given by

$$\bar{R} = \sum_{m=1}^{M} W_m x_m. \tag{S1}$$

For random variation in the rewards, one possibility is to assume additive variation, but we instead assume that R is log-normally distributed, preventing negative values. Thus,

$$R = \exp(\mu_R + z_R), \tag{S2}$$

with μ_R such that $\exp(\mu_R + \sigma_R^2/2) = \bar{R}$, with $\bar{R}$ from equation (S1), i.e. $\mu_R = \log(\bar{R}) - \sigma_R^2/2$, with, where z_R is normally distributed with mean zero and standard deviation σ_R. We might, for instance, have $\sigma_R = 0.1$, which is used in Figures 1 to 3 in the main text.

Our learning models, as described by equations (1, 2, 3) in the main text and

equations (S1, S2) here, are examples of action-value learning. One can view such learning as a modification of classical conditioning, making it applicable to instrumental conditioning (see sections 2.2 and 2.5 in Sutton and Barto (2018) for discussion of this learning approach). Note also that action-value learning can be regarded as a simplified version of the Sarsa algorithm, for cases where individuals do not use any sophisticated states and where each learning trial is a separate episode (terminology from Sutton and Barto 2018). We get a connection to the presentation in Sutton and Barto (2018) by assuming that the state in a trial is just the compound stimuli that are present in that trial, for the individual to choose between.

Learning rates

Rescorla-Wagner

The Rescorla-Wagner learning mechanism has learning rates that are constant in time. In our simulations, we assume that

$$\alpha_{mt} = \alpha_{\mathrm{RW}} \tag{S3}$$

for the learning rate for dimension m in trial t. Thus, we assume that the Rescorla-Wagner learning rate α_{RW} is constant, and thus independent of the stimulus dimension m and the trial t (we use $\alpha_{\mathrm{RW}} = 0.04$ in our simulations).

IDBD

The IDBD (Incremental Delta-Bar-Delta) learning algorithm was developed by Sutton (1992a), and is also described in Sutton (1992b) and by equations (1) and (3) in Mahmood et al. (2012). For clarity we write the prediction error from equation (2) in trial t as

$$\delta_t = R_t - Q_t, \tag{S4}$$

where Q_t is the estimated value from equation (3) for the chosen compound stimulus with stimulus components x_m. We write the estimated value as

$$Q_t = \sum_{m=1}^{M} x_m w_{m,t}. \tag{S5}$$

Using the order of updates for IDBD from Sutton (1992a, 1992b) and Mahmood et al. (2012), there are initial learning rates $\alpha_{m,1}$ and, to update the estimated values, we first update the learning rates as follows:

$$\alpha_{m,t+1} = \alpha_{m,t} \exp\bigl(\mu \delta_t x_m h_{m,t}\bigr), \tag{S6}$$

where μ is a meta learning rate, δ_t is from equation (S4), and $h_{m,t}$ is an additional quantity with starting value $h_{m,1} = 0$. The estimated value updates, corresponding to equation (1), are then

$$w_{m,t+1} = w_{m,t} + \alpha_{m,t+1} x_m \delta_t. \tag{S7}$$

The quantity $h_{m,t}$ is updated as follows

$$h_{m,t+1} = h_{m,t}\bigl[1 - \alpha_{m,t+1} x_m^2\bigr]^+ + \alpha_{m,t+1} x_m \delta_t. \tag{S8}$$

The notation $\bigl[X\bigr]^+$ means equal to X for positive X and zero otherwise.

For an intuitive interpretation, note the quantity h_m is a kind of memory or trace of changes to w_m, because those changes are given by $\alpha_{m,t+1} x_m \delta_t$. The exponent in equation (S6) contains the product of this trace with $\delta_t x_m$, which is proportional to the current change in w_m. Thus, if successive changes to w_m tend to be positively correlated, the learning rate α_m will increase, and similarly decrease for negative correlations.

Autostep

A problem with the IDBD algorithm, as pointed out by Mahmood et al. (2012), is that it is very sensitive to the exact value of the meta learning rate μ. To avoid this problem, Mahmood et al. (2012) introduced changes and obtained a more robust algorithm, which they called Autostep. It is called Autostep because the step size, or effective meta learning rate, is automatically adjusted to an appropriate value. The Autostep algorithm is given in Table 1 of Mahmood et al. (2012), and is as follows.

First set the two meta learning parameters μ and τ (we used $\mu = 0.2$ and $\tau = 100$ in our Autostep simulations). Then initialise $w_{m,1}$ and $\alpha_{m,1}$ (we used $w_{m,1} = 0$ and $\alpha_{m,1} = 0.04$) and set $v_{m,1} = 0$ and $h_{m,1} = 0$.

For each trial t we then have the following. The estimated values for compound

stimuli are computed from features x_m as

$$Q_t = \sum_{m=1}^{M} x_m w_{m,t}, \tag{S9}$$

just as previously. The learner chooses a compound stimulus using a soft-max rule, as in equation (4), and perceives a reward R. This gives a prediction error

$$\delta_t = R_t - Q_t, \tag{S10}$$

just as previously. Then compute the quantity

$$v_{m,t+1} = \max\big(|\delta_t x_m h_{m,t}|, v_{m,t} + \frac{1}{\tau}\alpha_{m,t} x_m^2 (|\delta_t x_m h_{m,t}| - v_{m,t})\big). \tag{S11}$$

If $v_{m,t+1} \neq 0$, compute

$$\alpha_{m,\text{temp}} = \alpha_{m,t} \exp\big(\frac{\mu \delta_t x_m h_{m,t}}{v_{m,t+1}}\big), \tag{S12}$$

otherwise use $\alpha_{m,\text{temp}} = \alpha_{m,t}$. Do this for each m and compute

$$S_\alpha = \max\big(\sum_{m=1}^{M} \alpha_{m,\text{temp}} x_m^2, 1\big). \tag{S13}$$

Use this S_α to normalise the learning rates

$$\alpha_{m,t+1} = \frac{\alpha_{m,\text{temp}}}{S_\alpha}. \tag{S14}$$

These are then used for the updates of the estimated values:

$$w_{m,t+1} = w_{m,t} + \alpha_{m,t+1} x_m \delta_t. \tag{S15}$$

Finally, the $h_{m,t}$ are updated as follows:

$$h_{m,t+1} = h_{m,t}\big[1 - \alpha_{m,t+1} x_m^2\big] + \alpha_{m,t+1} x_m \delta_t. \tag{S16}$$

The changes in Autostep from IDBD are, first, that the exponent in the update in equation (S6) is divided by the quantity $v_{m,t+1}$ in equation (S12), achieving a reasonable effective meta step size and, second, that the learning rates are normalised in equation (S14). See Mahmood et al. (2012) for more comments on the Autostep algorithm.
